# Strategies for Recurrent Atrial Fibrillation in Patients Despite Durable Pulmonary Vein Isolation

**DOI:** 10.3390/jcm14072250

**Published:** 2025-03-26

**Authors:** Jana Ackmann, Jonas Wörmann, Jakob Lüker, Friederike Pavel, Cornelia Scheurlen, Theodoros Maximidou, Jan-Hendrik van den Bruck, Jan-Hendrik Schipper, Daniel Steven, Arian Sultan

**Affiliations:** 1 Department of Electrophysiology, Heart Center of the University of Cologne, 50937 Cologne, Germany; 2 Department of Electrophysiology, Heart Center St. Georg, Asklepios, 20099 Hamburg, Germany

**Keywords:** atrial fibrillation, catheter ablation, ablation line, substrate modification, CFAE, PVI

## Abstract

**Background/Objectives**: Pulmonary vein isolation (PVI) is the cornerstone in the treatment of atrial fibrillation (AF). Despite initially successful PVI patients experience recurrence of AF potentially due to reconnection of pulmonary veins (PVs). However, a certain number of patients present with recurrent AF, despite durable PVI. The optimal ablation strategy for these patients has yet to be discerned. The aim of this study was to compare outcomes for different ablation strategies for recurrent AF despite persistent PVI. **Methods**: All redo procedures for the recurrence of atrial fibrillation from March 2018–May 2023 were analyzed. Only patients with proven durable PVI (entrance/exit block and high density (HD) mapping) who received linear ablation or CFAE (complex fractionated atrial electrogram)/low-voltage area ablation were included. Patients were excluded if re-PVI or ablation of atrial tachycardia (AT) was necessary. In all procedures, a 3D-HD map and radiofrequency ablation (RFA) were performed. The ablation strategy was at the operators’ discretion. Data from a routinely performed 12-month follow-up were obtained. **Results**: A total of 847 repeat ablation procedures for atrial arrhythmias were analyzed. In 170 (20.1%) procedures, all PVs were still isolated. Of these, 51 (30.0%) patients were excluded due to AT or because they did not receive further left atrial linear ablation or substrate modification. In total, 119 patients were included in the final analysis, and 71 out of 119 patients (59.7%) were male. The majority (89 patients, 74.8%) suffered from persistent AF. In 72 patients (60.5%), LA-scar (voltage < 0.4 mV) was detectable (81.9% persAF). The ablation strategies were either linear ablation (n = 55), a non-linear substrate modification strategy (CFAE ablation/ablation of low-voltage areas, n = 21) or a combination of both (n = 43). In the Kaplan–Meier analysis, none of the ablation strategies showed a significantly superior outcome. After 370.0 ± 144.9 days, 56.0% (48.1% vs. 61.9% vs. 62.8%, *p* = 0.3) were free from any arrhythmia. 15.4% vs. 9.5% vs. 9.3% developed an AT (*p* = 0.3). Left atrial dilatation correlated with recurrence of AF. **Conclusions**: In patients suffering from a recurrence of AF despite durable pulmonary vein isolation, different substrate modification strategies did not show any superiority for one or the other. Despite the necessity of additional ablation beyond PVI, the optimal ablation strategy has yet to be determined to improve the outcome of redo procedures.

## 1. Introduction

Pulmonary vein isolation (PVI) represents the foundation of atrial fibrillation (AF) treatment [1]. However, often more than one ablation procedure is necessary to sustain sinus rhythm. The progression of AF remodelling of the atrium can create an arrhythmogenic substrate [2], facilitating the transition from paroxysmal AF (PAF) to persistent AF (persAF) which complicates treatment. In repeat procedures for recurrence of AF, three-dimensional (3D) mapping is usually used to guide targeted ablation of arrhythmogenic substrate. Currently, radiofrequency ablation (RFA) is still the most widely used modality. The most common reason for recurrence of AF is reconnection of the pulmonary veins [3,4] which can be addressed by targeting gaps in PVI ablation lines. However, there are cases with recurrence of AF despite durable PVI [3] and the number might rise due to improved PVI protocols [5]. In these cases, ablation is more challenging [6], and the optimal ablation strategy is still unknown. So far, studies are sparse and have failed to establish a clearly superior ablation strategy [7]. Among the most common ablation strategies are the ablation of anatomical lines [8] and non-linear substrate-targeting approaches, including ablation of complex fractionated atrial electrograms (CFAE) [9] and low-voltage areas [10]. Today, none of these approaches is recommended [1], however real-world data indicate that applying these methods is still common practice.

This study was initiated to compare ablation of anatomical lines with non-linear substrate modification strategies (CFAE/low-voltage area ablation) in repeat ablation for AF in patients with durable PVI.

## 2. Materials and Methods

### 2.1. Study Design

We performed a retrospective cohort analysis of re-ablation procedures for AF between March 2018 and May 2023 at the University Hospital Cologne. Patients with durable PVI who received radiofrequency ablation with either ablation lines, substrate modification or both were included. Patients were excluded if they had pulmonary vein reconnection, documentation of AT, were younger than 18 years, or had not provided written informed consent. Data from routinely performed follow-up visits in our center including either Holter-ECG, 12-lead ECG, app-based telemonitoring or if applicable a device interrogation were analyzed. Recurrence of arrhythmia was defined by documentation of AF, AT or atrial flutter with a duration over 30 s.

### 2.2. Ablation Procedure

In all cases, informed consent of the patients was provided. Fentanyl, midazolam, and propofol were used to achieve deep analgo-sedation for each procedure. If patients were on vitamin K antagonists, those were continued and an INR of 2–3 was targeted for the ablation. Direct oral anticoagulants (DOACs) were discontinued either the day prior to ablation (in case of one daily dose) or the evening prior to ablation (in case of two daily doses). Patients who presented in AF or were not on continuous anticoagulation and had a CHA_2_DS_2_-VASc-Score of at least 2 received a transoesophageal echocardiogram (TOE) to rule out intracardiac thrombi [11]. The right femoral vein was the access site. Following a three-fold groin puncture a catheter (Dynamic XT™, large curve 4.0/Decapolar; Boston Scientific, Marlborough, MA, USA) was placed in the coronary sinus (CS), followed by transseptal puncture under fluoroscopic control (TSX™ fixed curve transseptal sheath; Boston Scientific, TSX™ transseptal needle; Boston Scientific, Marlborough, MA, USA). By weight-adjusted administration of unfractionated heparin (UFH), an activated clotting time (ACT) of over 300 s was targeted. Every 30 min ACT measurement was repeated and ACT adjusted UFH doses were injected.

A 3D-Mapping system (Ensite NavX/Ensite Precision/Ensite X; Abbott, Abbott Park, IL, USA or Carto; Biosense Webster, Irvine, CA, USA) and a multipolar mapping catheter (Advisor™ HD Grid Mapping Catheter, Sensor Enabled™; Abbott, LASSO^®^ Circular Mapping Catheter; Biosense Webster, Irvine, CA, USA or PENTARAY^®^ NAV ECO High Density Mapping Catheter; Biosense Webster, Irvine, CA, USA) were used in all procedures. Durable PVI was confirmed for all pulmonary veins (entrance-/exitblock, 3D-HD map). The ablation strategy was at the operator’s discretion. The ablation catheters utilized were contact force-measuring models, including the FlexAbility™ Ablation Catheter, Sensor Enabled™ (Abbott, Abbott Park, IL, USA), the TactiCath™ Contact Force Ablation Catheter, Sensor Enabled™ (Abbott, Abbott Park, IL, USA), and the THERMOCOOL SMARTTOUCH^®^ SF Catheter (Biosense Webster, Irvine, CA, USA). If typical atrial flutter was observed additional CTI ablation was performed. The groin access site was closed by a figure-of-eight suture. All patients received a compression bandage for a minimum of 6 h. Immediately after the procedure, 2 h after the procedure and the next morning all patients received a transthoracic echocardiogram (TTE) to exclude pericardial effusion. The procedure time was determined from the moment of groin puncture until the sheath was removed. ECG monitoring was continued for at least 24 h.

### 2.3. Statistical Analysis

Data acquisition was performed with a case report form using an electronic database (RedCap, Research Electronic Data Capture [12]). Statistical analysis was performed using Microsoft Excel for Mac (version 16.86, Microsoft Corporation, Redmond, WA, USA) and Prism 10 for MacOS (version 10.2.3, GraphPad Software, San Diego, CA, USA). Continuous variables are displayed as mean (±SD) and dichotomous variables as absolute values and percentages. A Gaussian distribution of continuous variables was evaluated with the Shapiro–Wilk test and analysis was carried out either with the one-way ANOVA if variables were normally distributed or otherwise with the Kruskal–Wallis test. As a Post Hoc test, Bonferroni was chosen. Dichotomous variables were evaluated using contingency tables and Fisher’s exact test. Survival analysis was conducted using Kaplan–Meier curves with the log-rank test, along with a multivariate Cox proportional hazards regression. *p*-values < 0.05 were considered as statistically significant.

## 3. Results

### 3.1. Patient Characteristics

**Figure 1:**
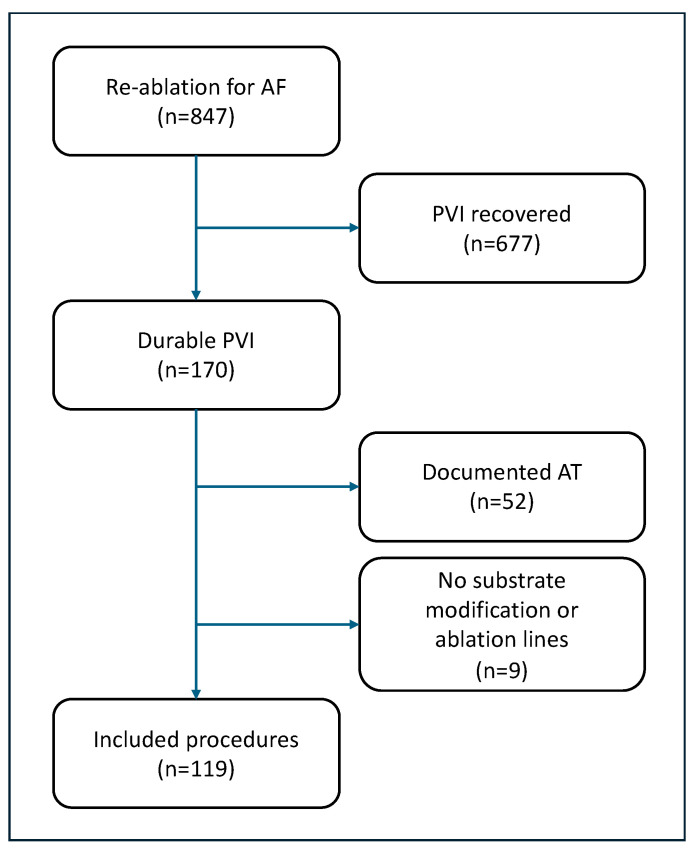
Decision path.

From March 2018 to May 2023, 847 patients underwent repeat catheter ablation (CA) for recurrence of AF. 170 patients (20.1%) showed durable PVI proven by either entrance or exit block and high-density (HD) voltage map. Of those, 42 patients were excluded due to documented AT and 9 were excluded because they did not receive further left atrial ablation lines or other substrate modification (Figure 1). In total, 119 (70%) patients with recurrence of AF were included. The majority of the included patients (74.8%) suffered from persAF.

Ablation strategies consisted of linear ablation for 55 (46.2%) patients (56.4% male; 67.1 ± 9.6 y), substrate modification (CFAE/low-voltage area ablation) for 21 (17.6%) patients (57.1% male; 69.4 ± 10.7 y), and a combination of both for 43 (36.1%) patients (65.1% male; 70.1 ± 8.8 y).

In all groups, baseline characteristics and comorbidities were comparable. Statistically, a preserved LVEF was marginally more prevalent in the linear ablation group (*p* = 0.049), and coronary artery disease was slightly less prevalent in patients receiving linear ablations (*p* = 0.02). In all three groups, more than two-thirds of patients suffered from persAF (69.1% vs. 71.4% vs. 83.7%, *p* = 0.2). The number of prior AF procedures was comparable (1.6 ± 0.8 vs. 1.9 ± 1.0 vs. 1.5 ± 0.7, *p* = 0.5). In the linear ablation group, one patient had received intra-operative ablation in addition to interventional PVI. The left atrial (LA) diameter was not significantly different between groups (41.4 ± 6.2 mm, 42.2 ± 4.4 mm, 43.3 ± 6.1 mm, *p* = 0.3).

Most patients were on beta-blockers (85.5% vs. 81.0% vs 83.7% *p* = 0.9) and anticoagulation (89.1% vs. 95.2% vs. 97.7%, *p* = 0.3). The use of antiarrhythmic drugs (AADs, 27.3% vs. 42.9% vs. 27.9%, *p* = 0.4) did not differ significantly between groups. Table 1 displays relevant baseline characteristics and comorbidities.

### 3.2. Ablation Strategies and Procedural Data

In 61% of patients, HD voltage mapping revealed an LA-scar (low-voltage < 0.4 mV). Patients with a scarred LA were more likely to suffer from persAF (80.9% vs. 63.8%, *p* = 0.032) and were more likely to undergo a combined approach (lines + substrate modification).

In the linear ablation group, the most prevalent ablation strategy was a box lesion followed by an anterior line and an LA roof line (Table 2). In the substrate modification group, all patients received ablation of CFAE/low-voltage areas and one patient received additional ablation of ectopic foci. In the combined strategies group, all patients received substrate modification and linear ablation lines and 4.7% received ablation of extrapulmonary ectopic foci that either occurred spontaneously or were provoked using isoprenaline. In this group, an anterior line was the most prevalent linear ablation strategy followed by an LA roof line and a box lesion (Table 2). If, in addition to recurrent AF, typical atrial flutter was documented, additional CTI-ablation was performed (12.7% vs. 38.1% vs. 20.9%, *p* = 0.1). In total, 42.7% of patients with persAF and 56.6% of patients with PAF underwent a linear ablation approach (*p* = 0.21). A CFAE/low-voltage area ablation strategy was applied to 16.9% of persAF patients and 20.0% of PAF patients (*p* = 0.78). A combined ablation approach was performed in 40.4% of persAF patients and 23.3% of PAF patients (*p* = 0.12). Overall, the ablation strategy did not differ significantly between persAF and PAF patients, although there was a trend toward a combined approach in persAF patients (Table A1 and Table A2).

Among groups the procedure time, fluoroscopy time and RFA duration were prolonged in the substrate modification group as compared to patients only receiving an anatomical linear approach (Table 3). Total RFA energy was lower in the linear ablation group than in the other groups.

### 3.3. Safety

Overall, complications were comparable between groups (*p =* 0.9, Table 4). Importantly, no ablation related death and no thromboembolic event occurred. There were two pericardial effusions in the linear ablation group and one in the substrate modification group that did not require pericardiocentesis. As for groin complications, one patient in the combined group developed both an AV-fistula and a pseudoaneurysm, and one patient in the linear ablation group developed a pseudoaneurysm. All were treated conservatively by groin compression. The AV-fistula was small and remained asymptomatic at follow-up. Furthermore, one aspiration and one sinus arrest were observed in the combined group.

### 3.4. Follow-Up

**Figure 2:**
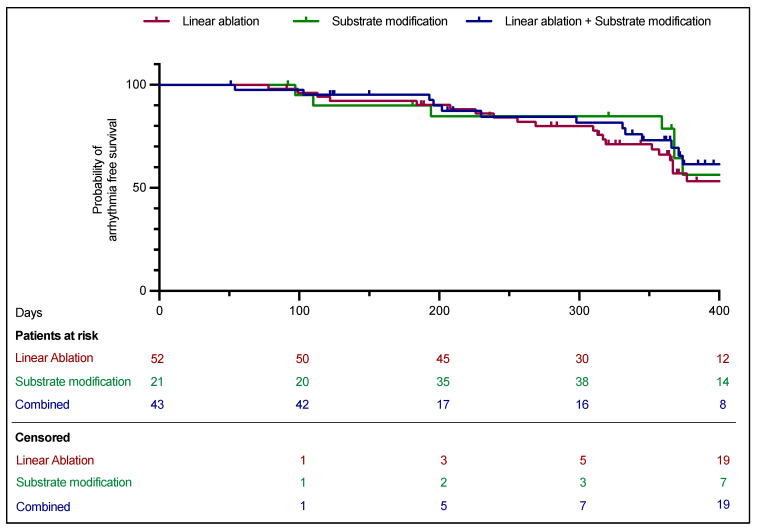
Kaplan–Meier analysis. Freedom of any atrial arrhythmia.

The mean follow-up was after 370.0 ± 144.9 days. For three patients receiving linear ablation, no follow-up was available (5.5%). In the survival analysis, the arrhythmia-free survival did not differ between groups (Figure 2, Log-rank *p* = 0.3) and was 56.0% (48.1% vs. 61.9% vs. 62.8%). Recurrence of AF occurred in 36.5% of patients in the linear ablation group, 28.6% in the substrate modification group, and 27.9% in the group with combined ablation strategy. Furthermore, 15.4% vs. 9.5% vs. 9.3% developed an AT (Log-rank *p* = 0.3).

A multivariable Cox proportional hazards regression was performed to evaluate the impact of clinical variables. Left atrial (LA) diameter was the only significant predictor of AF recurrence (HR 1.10, 95% CI 1.03–1.17, *p* = 0.002), indicating that a larger LA diameter was associated with an increased risk of recurrence. Other factors, including ablation strategy, age, sex, AF type, comorbidities (coronary artery disease, diabetes, hypertension, hyperlipoproteinemia, prior stroke/TIA), GFR, LA scar, LVEF, smoking and BMI, did not demonstrate a significant association with AF recurrence.

**Figure 3:**
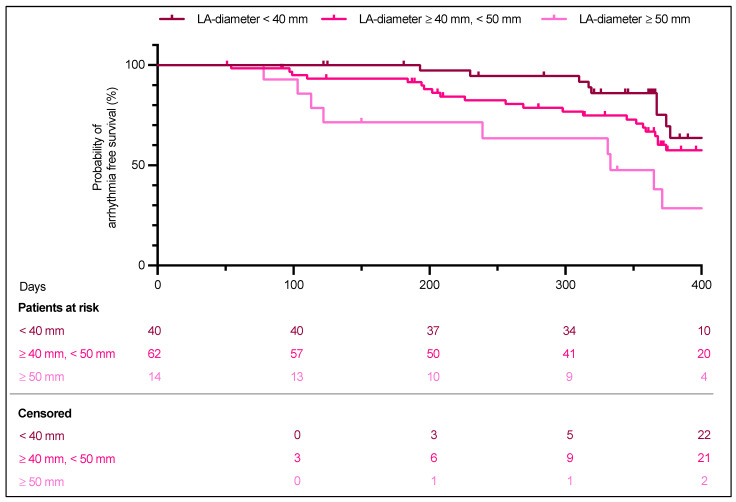
Kaplan–Meier analysis, Freedom of any atrial arrhythmia in patients with different LA-diameter. For two patients in the LA diameter < 40 mm group and for one patient in the ≥40 mm, <50 mm group no follow-up was available.

Given that LA dilation was associated with AF recurrence in the Cox regression, we conducted a Kaplan–Meier analysis to evaluate survival based on LA diameter. The analysis revealed that an enlarged LA diameter was linked to a shorter recurrence-free survival time (log-rank *p* = 0.0295, Figure 3).

## 4. Discussion

This retrospective analysis revealed comparable outcomes for different ablation strategies (anatomical ablation lines, CFAE/low-voltage area ablation and a combination of both) in patients undergoing repeat AF ablation for recurrence of AF despite durable PVI.

Durable PVI is the basis of successful AF-therapy. In the literature, a range of durable isolation of PVs of 5% [3,4] to 62% [5] in a more recent study was reported. In our study, in about 20% of analyzed procedures, the durable PVI of all four PVs was proven, which is in between of previously reported data. Remarkably, most patients suffered from persAF and 60.5% had proof of low-voltage areas in the LA.

Within groups ablation strategies were still heterogenous, e.g., with different sets of ablation lines. This might, on the one side, reflect the lack of general recommendations and on the other side reflect that decision-making was based on the individual substrate presented in the electro-anatomical mapping. Remarkably, a combined ablation strategy was chosen more often in patients with proof of low-voltage areas in the LA.

Lesion durability remains the key to ablation success. Although lesion sets were confirmed from endocardial in our study, a recent study by La Fazia et al. highlights the challenges of achieving transmural posterior wall isolation [13]. Their findings indicate that a 50 W power setting achieved epicardial isolation in 83.3%, whereas 40 W and 90 W settings failed to achieve epicardial isolation entirely, potentially leading to endo-epicardial dissociation and reentrant circuits. While the small sample size limits the study’s generalizability, we did not perform systematic epicardial validation, and undiagnosed epicardial gaps cannot be entirely ruled out. Further studies are needed to systematically assess lesion durability, particularly in terms of epicardial isolation, to determine whether improved techniques can enhance procedural success and reduce AF recurrence.

Linear ablation lines and substrate-based strategies (CFAE/low-voltage area ablation) are common ablation strategies, especially in repeat ablation for AF. For some subgroups, such as patients with mitral regurgitation and extensive substrate in the LA, additional strategies to PVI in the first procedure might improve outcomes [14]. However, those strategies are not, in general, recommended for the first procedure, since multiple studies failed to show a clear benefit in the general AF population [15,16,17] and extensive LA-ablation can create a new substrate for re-entrant circuits that increases the number of ATs [17,18,19,20].

There are only a few studies investigating AF patients with durable PVI. The PARTY-PVI study, a multicenter study comparing different ablation strategies for AF-ablation in patients with durable PVI [7] showed no significant difference between linear ablation, electrogram- (EGM-)based ablation, trigger-based ablation, PV-based ablation (more antral isolation of PV-isolation lines) and a mixed approach in recurrence of AF. Recurrence of atrial arrhythmia was 33.2% after 12 months which is slightly better than our outcome (44.0%). Of note, more patients included in our study suffered from persAF potentially explaining this outcome difference. In line with results from the PARTY-PVI-study, we found LA dilatation to be a predictor for a worse outcome. Another approach for AF-ablation is left atrial appendage (LAA) isolation which is due to the thromboembolic risk not frequently performed (in our study one patient received LAA isolation). This strategy was recently investigated in the ASTRO study for patients with durable PVI and failed to show a benefit [21].

Even though a benefit of linear ablation in addition to the first PVI [8] was demonstrated in single studies, this effect is not consistently reported. Verma et al. compared PVI alone with PVI + linear ablation and PVI + CFAE ablation, demonstrating no benefit of ablation beyond PVI [15]. Moreover, Vogler et al. found no superiority of a stepwise approach with PVI, CFAE ablation and linear ablation compared to PVI alone [22]. Estner et al. compared a PVI + CFAE approach with a PVI + linear ablation approach demonstrating similar arrhythmia-free survival. In that study in the PVI + linear ablation group, more patients experienced a recurrence of AF whereas in the PVI + CFAE group more patients experienced AT [23]. However, also linear ablation lines can cause an increase in ATs [24]. In our study, we could not find a statistically significant difference in AF and AT recurrence between groups.

Structural and functional remodelling plays a crucial role in AF maintenance. Fibrosis and electrical heterogeneity promote conduction slowing and reentrant circuits. In some studies, low-voltage area ablation had beneficial results [25,26], which are not consistent in the literature. Even though the DECAAF trial [27] confirmed an association of MRI-detected atrial fibrosis with AF recurrence, the MRI-guided ablation of low-voltage areas (DECAFF II) showed no benefit but a higher incidence of stroke and complications [28]. Therefore, it is not recommended to be performed in the current guidelines [1]. A direct comparison with our study is limited, as we did not use additional imaging to identify fibrotic areas. The VOLCANO trial [29] failed comparably to demonstrate the benefit of ablation of low-voltage areas in addition to the first PVI. This trial included exclusively PAF patients, which makes comparison with our cohort difficult. Remarkably, low-voltage areas were a predictor of a worse outcome and patients with low-voltage area ablation were more prone to AT. In contrast, in our study, we did not observe an increased AT incidence. Similarly, the STABLE-SR-II trial investigated low-voltage area ablation in persAF patients and found that, although the presence of low-voltage areas was associated with worse outcomes, additional ablation of those did not improve clinical success rates [30]. These findings suggest that the presence of low-voltage areas may reflect disease severity rather than serving as an effective ablation target. Consistent with this, our results showed no improved outcomes following substrate modification. LA dilatation, as an indicator of atrial remodelling, was associated with worse outcomes. However, in our study, Cox regression did not identify a significant association between AF type or LA scarring and AF recurrence, possibly due to limited sample size or unmeasured confounders. Given the correlation between LA diameter and AF recurrence, some patients with advanced atrial myopathy may no longer benefit from ablation. Even though LA diameter is a widely available and easily measurable parameter, it does not adequately quantify LA function. As a precise measure of functional impairment, LA strain has been shown to predict AF recurrence [31]. Notably, heart failure patients are particularly prone to atrial myopathy but, at the same time, may significantly benefit from sinus rhythm [32]. Therefore, for future studies, evaluating the efficacy of different strategies in patient subsets with and without atrial myopathy—assessed not only by LA diameter but also by LA strain as a functional parameter—will be of interest and may help to separate patients who can benefit from further ablation from patients with irreversible atrial myopathy.

Recurrence of AF despite durable PVI and a lack of success of the common ablation strategies suggests that additional mechanisms beyond fibrosis-related conduction block, which are potentially not fully understood, contribute to arrhythmia persistence. Autonomic influences and especially right atrial (RA) structures may serve as sources of non-PV triggers. Previous studies have identified the superior vena cava (SVC), interatrial septum, coronary sinus and right atrium (RA) as common sources of non-pulmonary vein triggers in AF patients [33] and have detected RA triggers in patients following initial PVI [34]. Notably, in older patients, right atrial triggers may predict AF independently of traditional left heart parameters [35]. Since we did not routinely screen for right atrial triggers in our cohort, this could explain why some patients did not respond to the applied left atrial ablation strategies. Moreover, it is known that the autonomic nervous system has an influence on AF and some studies showed that ablation of ganglionated plexi may increase freedom from atrial arrhythmia [36]. However, the clinical efficacy remains uncertain due to conflicting evidence, potential autonomic reinnervation and the absence of standardized techniques. Further mechanisms of AF focal drivers and rotors have been proposed [37]. However, ablation of those showed heterogeneous results and a lack of reproducibility [38]. Given the complexity of AF ablation beyond pulmonary veins, emerging ablation strategies using artificial intelligence (AI) may help optimize outcomes. Recently, a new AI-guided approach based on spatio-temporal dispersion mapping showed promising results with 88% AF freedom compared to 70% in the PVI-only group [39]. Its performance in patients with AF recurrence despite isolated PVs remains to be seen.

Ablation time, fluoroscopy time, and procedure time were longer in the substrate modification group even though the number of ablation points was comparable between groups. Therefore, a purely anatomical approach might be more practical and sufficient for most patients. Complications were not different between groups indicating comparable safety of the different approaches.

### Limitations

It is important to mention that this study has several limitations. First, this is a retrospective cohort study from a single center with a limited number of participants. Due to the retrospective design, the possibility of selection bias cannot be ruled out, especially in light of the finding that patients with a scarred LA received more often a combined ablation strategy. Furthermore, the ablation strategy was at the operator’s discretion and different sets of ablation lines and other substrate modification strategies were summarized in groups, potentially leading to misclassification bias. Residual confounding cannot be excluded, as unmeasured or unknown variables may have influenced the results. Moreover, no continuous rhythm monitoring was available, meaning that subclinical episodes of AF may have been missed potentially leading to an underestimation of AF recurrence and an increased risk of Type II error. 

To confirm the results of this study further research, preferentially large RCTs are necessary.

## 5. Conclusions

In patients experiencing recurrent AF despite durable pulmonary vein isolation, no substrate modification strategy has demonstrated clear superiority over others. While additional ablation beyond PVI is often required, the optimal approach for improving outcomes in redo procedures remains to be determined.

## Figures and Tables

**Table 1:** Baseline characteristics of the study population.

	Total	Ablation Strategy			*p*-Value
		Linear	Non-Linear	Combined	
Sex (male)	71/119 (59.7%)	31/55 (56.3%)	12/21 (57.1%)	28/43 (65.1%)	0.7
Age	68.6 ± 9.5	67.1 ± 9.6	69.4 ± 10.7	70.1 ± 8.8	0.2
BMI (kg/m^2^)	27.5 ± 4.6	27.4 ± 4.5	27.3 ± 4.2	27.6 ± 5.0	0.9
LVEF					
LVEF ≥ 50%	98/119 (82.4%)	48/55 (87.3%)	16/21 (76.2%)	34/43 (79.1%)	0.049
LVEF 40% to ≤50%	13/119 (10.9%)	4/55 (7.3%)	3/21 (14.3%)	6/43 (14.0%)	0.5
LVEF 30% to ≤40%	6/119 (5.0%)	2/55 (3.6%)	2/21 (9.5%)	2/43 (4.6%)	0.5
LVEF < 30%	2/119 (1.7%)	1/55 (1.8%)	0/21 (0%)	1/43 (2.3%)	1.0
PersAF	89/119 (74.8%)	38/55 (69.1%)	15/21 (71.4%)	36/43 (83.7%)	0.2
EHRA					
1	1/119 (0.8%)	0/55 (0%)	0/21 (0%)	1/43 (2.3%)	0.5
2a	7/119 (5.9%)	3/55 (5.5%)	2/21 (9.5%)	2/43 (4.7%)	0.8
2b	44/119 (37.0%)	23/55 (41.8%)	3/21 (14.3%)	18/43 (41.9%)	0.1
3	64/119 (53.8%)	28/55 (50.9%)	14/21 (66.7%)	22/43 (51.2%)	0.4
4	3/119 (2.5%)	1/55 (1.8%)	2/21 (9.5%)	0/43 (0%)	0.1
LA-diameter (mm)	42.2 ± 5.9	41.4 ± 6.2	42.2 ± 4.4	43.3 ± 6.1	0.3
Prior AF procedures	1.6 ± 0.8	1.6 ± 0.8	1.9 ± 1.0	1.5 ± 0.7	0.5
Arterial hypertension	90/119 (75.6%)	39/55 (70.9%)	17/21 (81.0%)	34/43 (79.1%)	0.7
Diabetes	17/119 (14.3%)	8/55 (14.5%)	4/21 (19.0%)	5/43 (11.6%)	0.7
Hyperlipoproteinemia	45/119 (37.8%)	23/55 (41.8%)	9/21 (42.9%)	13/43 (30.2%)	0.4
Coronary artery disease	21/119 (17.6%)	4/55 (7.3%)	5/21 (23.8%)	12/43 (27.9%)	0.02
Aktive Smoking	5/119 (4.2%)	4/55 (7.3%)	0/21 (0%)	1/43 (2.3%)	0.5
History of Smoking	22/119 (18.5%)	8/55 (14.5%)	4/21 (19.0%)	10/43 (23.3%)	0.6
History of stroke or TIA	9/119 (7.6%)	5/55 (9.0%)	0/21 (0%)	4/43 (9.3%)	0.4
GFR (mL/min; CKD-EPI)	70.2 ± 19.4	71.7 ± 18.3	70.2 ± 23.8	68.3 ± 18.7	0.5
CHA_2_DS_2_-VASc-Score	2.8 ± 1.5	2.5 ± 1.5	2.9 ± 1.4	3.1 ± 1.3	0.1
Medication					
Beta-blockers	100/119 (84.0%)	47/55 (85.5%)	17/21 (81.0%)	36/43 (83.7%)	0.9
AADs	36/119 (30.3%)	15/55 (27.3%)	9/21 (42.9%)	12/43 (27.9%)	0.4
Anticoagulation	111/119 (93.3%)	49/55 (89.1%)	20/21 (95.2%)	42/43 (97.7%)	0.3

**Table 2:** Ablation strategies.

	Ablation Strategy		
	Linear	Substrate Modification	Combined
Linear ablation strategies			
LA Isthmus	3/55 (5.5%)		2/43 (4.7%)
LA Roof	16/55 (29.1%)		17/43 (39.5%)
Inferior Line	0/55 (0%)		4/43 (9.3%)
Anterior line	15/55 (27.3%)		25/43 (58.1%)
Box	37/55 (67.3%)		16/43 (37.2%)
LAA	0/55 (0.0%)		1/43 (2.3%)
Non-linear ablation strategies			
CFAE		15/21 (71.4%)	26/43 (60.5%)
Ablation of low-voltage areas		6/21 (28.6%)	17/43 (39.5%
Ectopic foci		1/21 (4.8%)	2/43 (4.7%)

**Table 3:** Procedural data.

	Total	Ablation Strategy			*p*-Value
		Linear	Substrate Modification	Combined	
LA-scar	72/119 (60.5%)	27/55 (49.1%)	12/21 (57.1%)	33/43 (76.7%)	0.0180
Procedure time (min)	155.7 ± 60.0	144.1 ± 65.2	175.0 ± 55.4	161.3 ± 52.8	0.0176
Fluoroscopy dose (mGy/cm^2^)	6084.8 ± 1412.3	4228.0 ± 4255.3	6724.3 ± 10494.8	8147.6 ± 8340.1	0.2
Fluoroscopy time (min)	13.0 ± 10.4	9.8 ± 8.4	17.9 ± 11.5	14.7 ± 11.0	0.0024
RFA impulses	76.3 ± 54.6	64.9 ± 30.5	69.2 ± 37.1	94.3 ± 77.1	0.2
RFA duration (s)	2352.0 ± 1521.3	1981.5 ± 1412.6	3174.3 ± 1636.2	2443.5 ± 1469.8	0.0017
Total energy (J)	82,043.2 ± 47,954.6	68,239.6 ± 41,766.8	98,242.7 ± 54,144.8	92,164.5 ± 48,556.0	0.0033

**Table 4:** Complications.

	Total	Ablation Strategy			*p*-Value
		Linear	Substrate Modification	Combined	
Stroke/TIA	-	-	-	-	-
Pericardial effusion without tamponade	2/119 (1.7%)	2/55 (3.6%)	1/21 (4.8%)	-	0.4
Pericardial tamponade	-	-	-	-	-
Severe groin hematoma requiring further intervention	-	-	-	-	-
Aneurysma spurium	2/119 (1.7%)	1/55 (1.8%)	-	1/43 (2.3%)	0.5
AV-fistula	1/119 (0.8%)	-	-	1/43 (2.3%)	0.5
Aspiration	1/119 (0.8%)	-	-	1/43 (2.3%)	0.5
Sinus arrest	1/119 (0.8%)	-	-	1/43 (2.3%)	0.5
Esophagoatrial fistula	-	-	-	-	-
Phrenic nerve palsy	-	-	-	-	-
Total	7/119 (5.9%)	3/55 (5.4%)	1/21 (4.8%)	4/43 (9.3%)	0.9

## Data Availability

The data presented in this study are available on request from the corresponding author due to privacy considerations.

## Abbreviations

The following abbreviations are used in this manuscript:

AAD	Anti-arrhythmic drug
AI	Artificial intelligence
ACT	Activated clotting time
AF	Atrial fibrillation
AT	Atrial tachycardia
BMI	Body mass index
CA	Catheter ablation
CFAE	Complex fractionated atrial electrograms
CS	Coronary sinus
CTI	Cavo-tricuspid isthmus
DOAC	Direct oral anticoagulant
ECG	Electrocardiogram
EGM	Electrogram
EHRA	European heart rhythm association
GFR	Glomerular filtration rate
HD	High density
INR	International normalized ratio
LA	Left atrium
LAA	Left atrial appendage
LVEF	Left ventricular ejection fraction
MRI	Magnet resonance imaging
PAF	Paroxysmal atrial fibrillation
persAF	Persistent atrial fibrillation
PV	Pulmonary vein
PVI	Pulmonary vein isolation
RA	Right atrium
RFA	Radio frequency ablation
TIA	Transitory ischemic attack
TOE	Transoesophageal echocardiogram
TTE	Transthoracic echocardiogram
UFH	Unfractionated heparin

## Appendix A

**Table A1:** Ablation strategies in persAF patients.

	Ablation Strategy		
	Linear	Substrate Modification	Combined
Number of patients	38/89 (42.7%)	15/89 (16.9%)	36/89 (40.4%)
Linear ablation strategies			
LA Isthmus	2/38 (5.3%)		2/36 (5.6%)
LA Roof	9/38 (23.7%)		14/36 (38.9%)
Inferior Line	0/38 (0%)		3/36 (8.3%)
Anterior line	14/38 (36.8%)		22/36 (61.1%)
Box	27/38 (71.1%)		14/36 (38.9%)
LAA	0/38 (0%)		1/36 (27.8%)
Non-linear ablation strategies			
CFAE		10/15 (66.7%)	22/36 (61.1%)
Ablation of low-voltage areas		5/15 (33.3%)	14/36 (38.9%
Ectopic foci		1/15 (6.7%)	2/36 (5.6%)

**Table A2:** Ablation strategies in PAF patients.

	**Ablation Strategy**		
	**Linear**	**Substrate Modification**	**Combined**
Number of patients	17/30 (56.6%)	6/30 (20.0%)	7/30 (23.3%)
Linear ablation strategies			
LA Isthmus	1/17 (5.9%)		0/7 (4.7%)
LA Roof	7/17 (41.2%)		3/7 (42.8%)
Inferior Line	0/17 (0%)		1/7 (14.3%)
Anterior line	1/17 (5.9%)		3/7 (42.8%)
Box	10/17 (58.8%)		2/7 (28.6%)
LAA	0/17 (0%)		0/7 (20%)
Non-linear ablation strategies			
CFAE		5/6 (83.3%)	4/7 (57.1%)
Ablation of low-voltage areas		1/6 (16.7%)	3/7 (42.9%
Ectopic foci		0/6 (0%)	0/7 (0%)
